# Mid-upper-arm circumference based case-detection, admission, and discharging of under five children in a large-scale community-based management of acute malnutrition program in Nigeria

**DOI:** 10.1186/s13690-018-0266-4

**Published:** 2018-04-09

**Authors:** Stanley Chitekwe, Sibhatu Biadgilign, Assaye Tolla, Mark Myatt

**Affiliations:** 1 United Nations Children’s Fund (UNICEF), Nigeria country office, UN House, Plot 617/618 Central Area District Diplomatic Zone, Garki, Abuja, P M B 2851 Nigeria; 2 Consultant Epidemiologist, Brixton Health, Llawryglyn, Wales UK

**Keywords:** Case-detection, MUAC, Admission, Discharging, CMAM, Nigeria

## Abstract

**Background:**

Severe acute malnutrition (SAM) threatens the lives of millions of children worldwide particularly in low and middle-income countries (LMICs). Community-based management of acute malnutrition (CMAM) is an approach to treating large numbers of cases of severe acute malnutrition (SAM) in a community setting. There is a debate about the use of mid-upper arm circumference (MUAC) for admitting and discharging SAM children. This article describes the experience of using MUAC for screening, case-finding, referral, admission, and discharge in a large-scale CMAM program delivered through existing primary health care facilities in Nigeria.

**Methods:**

Over one hundred thousand (*n* = 102,245) individual CMAM beneficiary records were collected from two of the eleven states (i.e. Katsina and Jigawa) that provide CMAM programming in Nigeria. The data were double entered and checked using EpiData version 3.2 and analyzed using the R language for data-analysis graphics.

**Results:**

The median MUAC at admission was 109 mm. Among admissions, 37.4% (38,275) had a comorbidity recorded at admission and 7.4% (7537) were recorded as having developed comorbidity during the treatment. Analysis in the better performing state program in the most recent year for which data were available found that 87.1% (*n* = 13,273) of admitted cases recovered and were discharged as cured, 9.2% (*n* = 1396) defaulted and were lost to follow-up, 2.9% (*n* = 443) were discharged as non-recovered, 0.7% (*n* = 104) were transferred to inpatient services, and 0.2% (*n* = 27) were known (died, to be dead or to have passed) during the treatment episode. The program met SPHERE minimum standards for treatment outcomes for therapeutic feeding programs. Factors associated with negative outcomes (default, non-recovery, transfer, and death) were distance between home and the treatment center; lower MUAC, diarrhea and cough at admission; or developing diarrhea, vomiting, fever, or cough during the treatment episode.

**Conclusions:**

This study confirms that MUAC can be used for both admitting and discharging criteria in CMAM programs with MUAC < 115 mm for admission and MUAC > = 115 mm or at discharge (a higher discharge threshold could be used). Long distances between home and treatment centers, lower MUAC at admission, or having diarrhea, vomiting, fever, or cough during the treatment episode were factors associated with negative outcome. Providing CMAM services closer to the community, using mobile and / or satellite clinics, counseling of mothers by health workers to encourage early treatment seeking behavior, and screening of patients at each patient visit for early detection and treatment of comorbidities are recommended.

## Background

Acute malnutrition threatens the lives of millions of children globally and the risk of dying is highest among severely malnourished children [1]. Children aged under five years with severe acute malnutrition (SAM) in Africa have high mortality rates without effective treatment [2]. Several Sub-Saharan Africa countries experience chronic food insecurity and recurrent drought and hunger that lead to inadequate dietary intake, in terms of both quality and quantity, to meet the nutritional needs of children [3]. It was estimated in 2005 that the proportion and number of severely wasted children in developing countries was 3.5% (95% CI: 1.8%–5.1%) and 19.3 (95% CI: 10.0–28.6) million children respectively. In addition to this, an estimated 449,160 child deaths (4.4% of all deaths) in children aged under 5 years were contributed to by severe wasting [4].

Community management of acute malnutrition (CMAM) is a relatively recent approach to manage SAM cases which aims to maximize program coverage while maintaining quality of care [5]. The aim of CMAM programming is to treat severe malnutrition and reduce mortality. Different anthropometric indicators have been used for identifying, referring, admitting, and discharging children in nutrition programs treating SAM. Currently, the World Health Organization (WHO) and United Nation Children Fund (UNICEF) recommends the use of MUAC less than 115 mm, the presence of bilateral pitting edema, or weight-for-height z-score (WHZ) below − 3 (only where the use of WHZ is feasible) as independent criteria for diagnosing and admitting SAM children aged between 6 and 59 months [6]. According to the recent Nigeria National Nutrition and Health Survey in 2014, the national prevalence of SAM in children aged between 6 and 59 months using the mid upper arm circumference (MUAC) case definition of MUAC < 115 mm and / or bilateral was 0.9% (95% CI = 0.7, 1.0) [7].

In large nutrition programs covering wide geographical areas and providing treatment at primary level facilities, as is the case in Nigeria, the use of MUAC for SAM diagnosis provides a simple, acceptable, fast, sensitive, specific, and low-cost indicator that can be measured by community volunteers and screening teams in the field [8]. In LMICs, MUAC has been shown to be the best indicator for screening and detection of malnutrition in a community [8]. In 2013 the WHO revised its recommendations to use WHZ ≥ − 2 (for cases admitted using WHZ), mid-upper-arm circumference ≥ 125 mm (for cases admitted using MUAC), or the absence of bilateral pitting oedema for at least 2 weeks (for cases admitted with edema) for discharging children from SAM treatment. Screening, admission, and discharge using WHZ is cumbersome requiring two or three trained staff and expensive equipment that is not part of essential clinic supply packs with measurements and calculations that are prone to error [9, 10] and height measurement and W/H calculation are not part of integrated management of childhood illness (IMCI) training. This article describes the experience of using MUAC for screening, case-finding, referral, admission, and discharge in a large-scale CMAM program delivered through existing primary health care facilities and has additional value in that it describes a large national program delivered by the statutory health sector when most CMAM reports are from small scale emergency programs run by NGOs.

## Methods

### Study setting and context

**Fig. 1:**
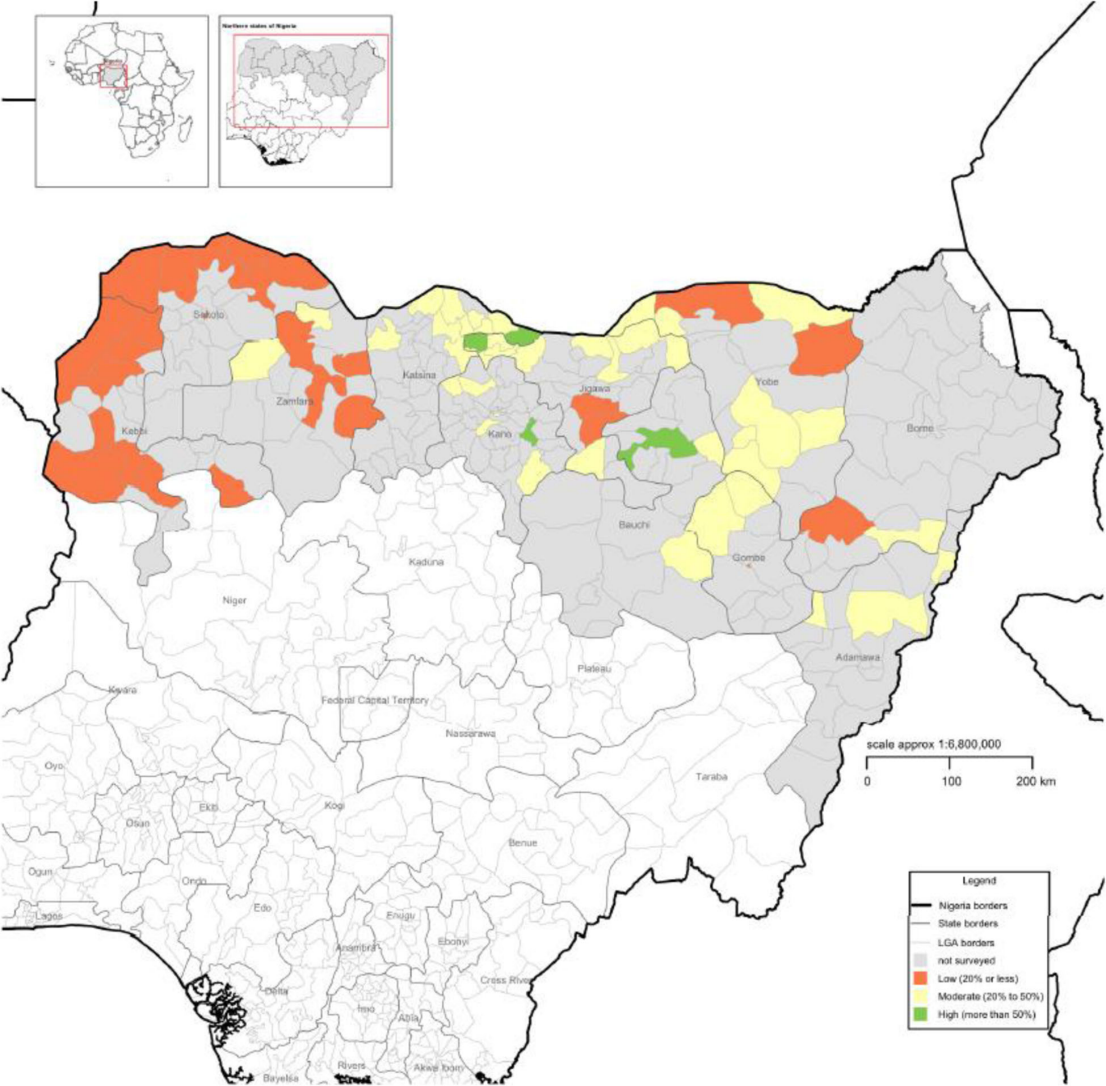
Map of Nigeria Outpatient Therapeutic Program operational area

The Nigerian CMAM program was started in 2009 in two states of northern Nigeria. Training for inpatient management of SAM started in the first quarter of 2009 followed by a pilot implementation of the CMAM approach in the second quarter of 2009 [11]. Figure 1 shows the map of Nigeria CMAM program.

Over one hundred thousand (*n* = 102,245) individual CMAM beneficiary records were collected from two of the 11 states that provide CMAM programming in Nigeria (i.e. Katsina and Jigawa states). These two states were selected because of the large number of admissions to CMAM sites in these states. Data for all children admitted to the CMAM program from 2010 (March 2010 for Jigawa and July 2010 for Katsina) to December 2013 were extracted from beneficiary record cards, copied onto data collection sheets, and entered into a purpose-designed database system. An analysis of the study dataset as being representative of CMAM programming in two states of Nigeria over the years 2010–2013 was performed.

### Operational definitions

The national guideline states that Cured as those who recovered/ meet the discharge criteria, defaulter means absent during three consecutive visits (declared defaulter at third absence), death as died during treatment in outpatient therapeutic program (OTP) program and non-recovered as those who did not meet the discharge cured criteria after four months in treatment. The health care workers are the one who confirmed the defaulter and death cases based on the national guideline/ protocol [11]. In this study, we define the following parameters used as follows. Weight gain (kg) as the difference between last recorded weight – admission weight; weight velocity (g/kg/day) defined as 1000 × last recorded weight − admission weight / last recorded weight × number of visits × 7; proportional weight gain defined as last recorded weight − admission weight / admission weight; and MUAC gain (mm) defined as last recorded MUAC – admission MUAC.

### Admission and discharging criteria

All the children admitted in CMAM program had weight, height, MUAC and bilateral pitting oedema measured / checked at the time of admission. Admission, treatment, and discharge followed the Nigerian National Guidelines for treating SAM cases [12]. The criteria for admission into the outpatient therapeutic program (OTP) were MUAC < 115 mm, or bilateral pitting oedema (+ or ++), or WHZ < − 3, passing the appetite test for the ready-to-use therapeutic food (RUTF), and presenting with no medical complications (i.e. vomiting, hyperthermia, hypothermia, lower respiratory tract infection / dyspnea, severe anemia, extensive skin lesions, unconsciousness, lethargy, dulled sensorium, hypoglycemia, convulsions, severe dehydration, or generalized (+++) edema) requiring inpatient treatment. All health facilities used MUAC and oedema admission criteria. The admission and discharge criteria recommended for each type of admission (i.e. MUAC, edema, WHZ) in the Nigerian CMAM program are shown in Table 1.

**Table 1:** Admission and discharge criteria (i.e. for patients discharged as cured) used in the Nigerian outpatient therapeutic program

Admission criteria	Discharge criteria
MUAC < 115 mm	MUAC > 115 mm and no edema, evidence of sustained weight gain, and clinically well^a^
Bilateral pitting edema	MUAC > 115 mm and no oedema for two consecutive visits (i.e. at least two weeks), and clinically well
WHZ < − 3	MUAC > 115 mm and WHZ > − 2 and no oedema for two consecutive visits, and clinically well

^a^This differs from the WHO recommendation of MUAC ≥125 mm. This was considered acceptable as the program is delivered in local primary healthcare centres and caregivers were instructed to return to the clinic of the child became ill or did not continue to gain weight after discharge

### Data management and analysis

To minimize data processing errors during coding and at data entry level, an extensive checking of data on the beneficiary record cards and on the computer -based methods was performed. In addition to this, manual checking was performed on all records cards from CMAM sites in the two selected states. Data were extracted and double-entered and validated at central level using a purpose designed database created using EpiData v3.2 software [13] by a motivated and experienced team of twenty data-entry clerks. Data were entered daily and checked as it was entered using legal value, range, and between-field consistency checks. These checks were repeated on all data in batch mode before data analysis. At each stage, identified errors were fixed by reference to original beneficiary record cards. Clearly erroneous data that could not be fixed were censored (i.e. marked as “missing”). Records were organized by CMAM site and missing key data items including missing CMAM site codes were recorded. Exit categories were checked and correctly recorded when not clearly recorded on the beneficiary record card, interactive checks for range and legal values were used to detect and correct problems as data were entered. These errors may arise from an invalid value recorded on the beneficiary record card or as a mistake during data entry by a data-entry clerk and 10 % of records (randomly selected on an ongoing basis) were double checked to verify the correctness of data. Two data-entry clerks with unacceptable rates of error were replaced during data-entry process and all records were re-entered by other data-entry clerks.

The data was analyzed using the R language for data-analysis and graphics version 3.1.0 [14]. Descriptive and inferential analyses were performed. The inferential analysis concentrated on identifying factors associated with different treatment outcomes. Association was decided by both *statistical significance* (i.e. *p* < 0.05) and by *substantive significance* [15]. Substantive significance was decided by examining effect sizes and comparing these against a standard *(see* Table 2) [15].

**Table 2:** Post-test effect sizes used to identify associations with substantive significance

Factor	Outcome	Test	Effect size	Standard^a^
Categorical	Categorical	Chi-square Risk ratio (RR) ≠ 1	Risk difference (RD)	|RD| > 5%
Categorical	Continuous	Kruskal-Wallis H	Cohen’s *d*^b^	|*d*| > 0.20
Continuous	Continuous	Wald type test for *β* coefficient = 0	Pearson’s |*r*^c^| > 0.10	|*r*^*c*^| > 0.10

^a^Standards were applied post-test (i.e. only for associations with *p* < 0.05)

^b^Difference between means divided by the pooled standard deviation [30]

^c^Pearson product moment correlation coefficient

This two-stage process was adopted in order to avoid the problem of *false discovery* that often occurs when analyzing very large samples (i.e. when even very small differences are statistically significant) using statistical significance tests alone [15].

Variables associated with negative treatment outcomes were entered a multivariate logistic regression model. A backwards stepwise elimination procedure was used to remove non-significant variables (i.e. *p* ≥ 0.05) from the model. At each step, the variable with the largest *p*-value ≥0.05 was removed from the model and the model re-estimated. This process continued until all non-significant variables had been eliminated.

## Results

A total of 102,245 cases of admitted children were recorded in the database. Among the total admitted children, 48.2% (*n* = 49,240) were males, 46.1% (*n* = 47,137) were females (and 5.7% (*n* = 5868) of beneficiary cards had no sex recorded). The median age at admission was 13 months. The sample is described in Table 3*.*

**Table 3:** Characteristics of the sample of admissions from the Nigeria outpatient therapeutic program (2010 to 2013)

Attribute	Details	Number	Percentage
Sample size	Number of children	102,245	100.00%
Sex	Males	49,240	48.2%
	Females	47,137	46.1%
	Missing	5868	5.7%
Age (year-centered age-group) at admission (months)^a^	[6,17] centred at 1 year	60,750	59.4%
	(17,29] centred at 2 years	32,170	31.5%
	(29,41] centred at 3 years	2520	2.4%
	(41,53] centred at 4 years	306	0.3%
	(53,59] centred at 5 years	56	0.1%
Age at admission (months)	Median age at admission	13	
Time to travel (hours)^a^	[0,0.5]	18,968	18.5%
	(0.5,1]	20,870	20.4%
	(1,2]	14,503	14.2%
	(2,3]	6348	6.2%
	(3,4]	1874	1.8%
	(4,5]	703	0.7%
	(5,6]	314	0.3%
	(6,7]	155	0.1%
	Missing	38,510	37.7%

^a^Intervals (ranges) are expressed in ISO 31–11 form. The form (a,b] expresses a < x ≤ b

The form [a,b] expresses a ≤ x ≤ b

**Fig. 2:**
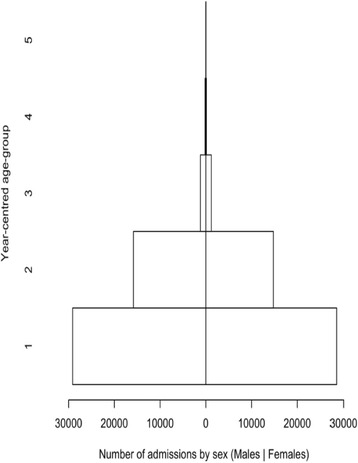
Age at admission for the cohort of children in Nigeria Outpatient Therapeutic Program (2010 to 2013)

**Fig. 3:**
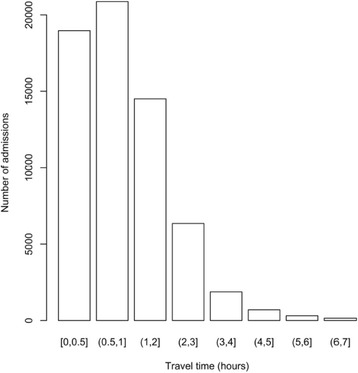
Histogram showing travel times in hours by the clients in Nigeria Outpatient Therapeutic Program (2010 to 2013)

**Fig. 4:**
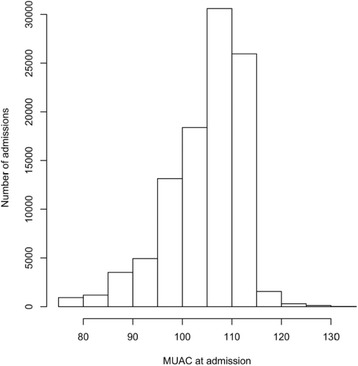
Histogram describing mid-upper arm circumference at admission for severe acute malnutrition cases in Nigeria Outpatient Therapeutic Program (2010 to 2013)

Figures 2, 3, and 4 show age at admission by sex, time to travel (in hours) from home to the program site, and MUAC at admission for SAM cases in the patient cohort of children. The majority (95.1%), *n* = 97,239) of the children were admitted using MUAC alone. The median MUAC at admission was 109 mm. 37.4% (*n* = 38,275) of admissions had a comorbidity recorded at admission and 7.4% (*n* = 7537) were recorded as having developed a comorbidity during the treatment episode [Table 4]. Lengths of stay, weight gain, weight velocity, proportional weight gain, and MUAC gain for each outcome (i.e. default, non-recovered, recovered (cured), transfer, and died) are summarized in Tables 5 and 6*.*

**Table 4:** Description of the cohort at admission (admission criteria, anthropometry, and comorbidity) in the sample of admissions from the Nigeria Outpatient Therapeutic Program (2010 to 2013)

Attribute	Details	Number	Percentage
Admission criteria	MUAC only	97,239	95.1%
	MUAC with oedema	1417	1.4%
	Oedema only	180	0.2%
	Other	3409	3.3%
MUAC at admission (mm)	Minimum	75.0	
	Lower quartile	102.0	
	Median	109.0	
	Mean	106.1	
	Upper quartile	111.0	
	Maximum	135.0	
	Missing	1568	1.5%
Weight-for-age z-score at admission	Minimum	−8.413	
	Lower quartile	−5.005	
	Median	−4.272	
	Mean	−4.288	
	Upper quartile	−3.583	
	Maximum	−0.085	
	Missing	14,022	13.7%
Comorbidity at admission	Any comorbidity	38,275	37.4%
	Diarrhoea	25, 011	24.5%
	Vomiting	13,768	13.5%
	Fever	20,893	20.4%
	Respiratory illness	14,288	14.0%
Comorbidity during the treatment episode^a^	Any comorbidity	7537	7.4%
	Diarrhoea	3993	3.9%
	Vomiting	1481	1.4%
	Fever	3755	3.7%
	Respiratory illness	2043	2.0%

^a^Reported only for cases with more than a single visit

**Table 5:** Outcomes of treatment in the sample of admissions from the Nigeria Outpatient Therapeutic Program (2010 to 2013)

Attribute	Details	Number	Percentage
Outcome (type of exit)	Defaulted	20,229	19.8%
	Non-recovered	8763	8.6%
	Recovered	72,463	70.9%
	Transferred	507	0.5%
	Died	283	0.3%
	Negative outcomes^a^	29,782	29.1%
Weight gain (kg)^b^	Minimum	−1.20	
	Lower quartile	0.50	
	Median	1.10	
	Mean	1.12	
	Upper quartile	1.60	
	Maximum	3.90	
Weight velocity (g/kg/day)^c^	Minimum	−4.86	
	Lower quartile	2.04	
	Median	3.44	
	Mean	3.63	
	Upper quartile	5.08	
	Maximum	13.9	
Proportional weight gain^d^	Minimum	−18.3%	
	Lower quartile	0.09	
	Median	0.18	
	Mean	0.20	
	Upper quartile	0.29	
	Maximum	0.84	
MUAC gain (mm)^e^	Minimum	−10.0	
	Lower quartile	8.00	
	Median	14.0	
	Mean	14.0	
	Upper quartile	20.0	
	Maximum	39.0	
Length of stay (visits)^f^	Minimum	2.00	
	Lower quartile	5.00	
	Median	7.00	
	Mean	6.80	
	Upper quartile	8.00	
	Maximum	27.0	

^a^All non-recovered cases (default, non-recovery, transfer, and death)

^b^Oedematous cases, cases with a single visit, and cases with extreme values censored (*n* = 89,165)

^c^Oedematous cases, cases with a single visit, and cases with extreme values censored. (*n* = 87,343)

^d^Oedematous cases, cases with a single visit, and cases with extreme values censored (*n* = 89,066)

^e^Cases with extreme values censored. Analysis is for *n* = 89,047 cases

^f^Recovered cases only (*n* = 72,463)

**Table 6:** Length of stay, weight gain, weight velocity, proportional weight gain, and mid-upper arm circumference gain by outcome in the sample of admissions from the Nigeria Outpatient Therapeutic Program (2010 to 2013)

Outcome	Length of stay (weeks)^a^	Weight gain (kg)^a^	Weight velocity (g/kg/day)^a^	Proportional weight gain^a^	MUAC gain (mm)^a^
Default	1 (1;5)	0.7 (0.2;1.2)	3.1 (1.3; 5.0)	10% (0.0; 0.2)	9 (3;15)
Non-recovered	4 (2;7)	0.3 (0.0;0.6)	1.6 (0.0; 3.0)	5% (0.0; 0.1)	3 (0;8)
Recovered	7 (5;8)	1.1 (0.5;1.6)	3.4 (2.0, 5.0)	20% (0.1; 0.3)	14 (8;20)
Transferred	7 (4;10)	0.7 (0.2;1.3)	2.4 (0.9; 4.0)	10% (0.0; 0.2)	8 (1;15)
Died	4 (2;6)	0.3 (−0.1;0.9)	1.9 (0.0;4.3)	5%(−0.0; 0.15)	4 (0;10)

^a^Results are presented as median (inter-quartile range)

**Fig. 5:**
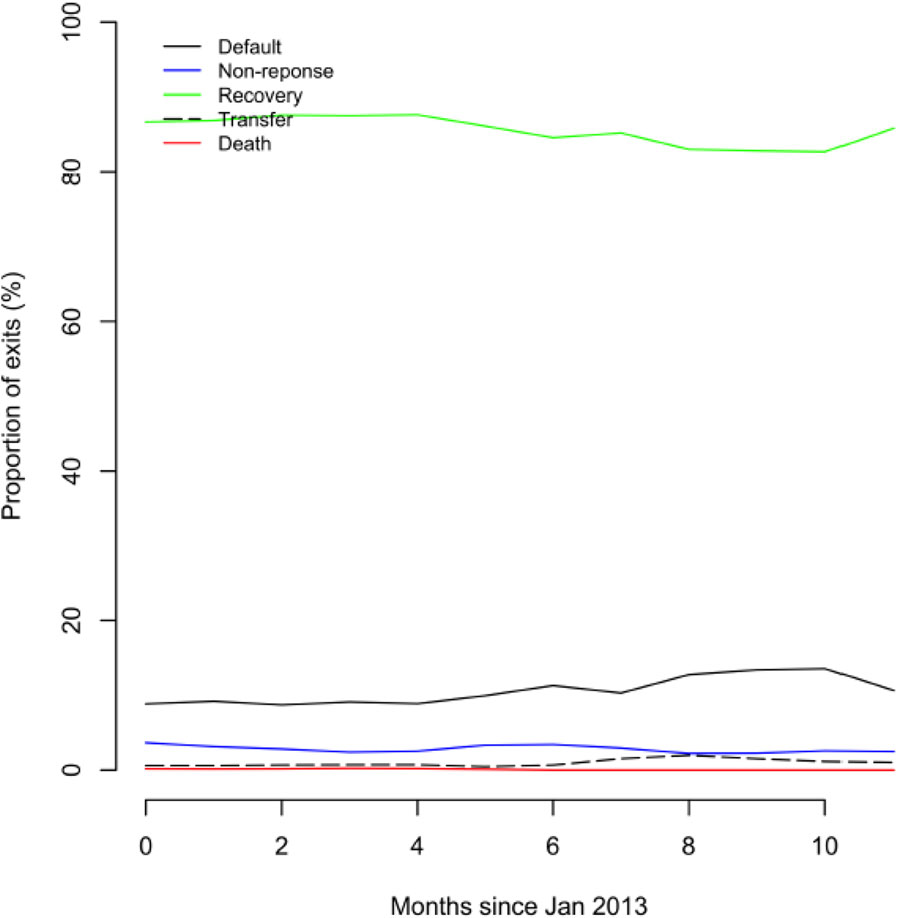
Outcomes (as proportions of all exits) in the sample of admissions from the Nigeria Outpatient Therapeutic Program (2013)

A separate analysis for one of the states for the year of 2013 was performed. This analysis is for the better performing state program in the most recent year for which data were available. This gives some indication of how well a large-scale CMAM program can perform in a given time. Such an analysis would not be appropriate for a small, short-term, and vertical NGO-delivered humanitarian and/or emergency program but may be appropriate for a large-scale, longer-term program delivered by the statutory sector as an extension to IMCI programming. This analysis found 87.1% (*n* = 13,273) of admitted cases recovered and were discharged as cured, 9.2% (*n* = 1396) defaulted and were lost to follow-up, 2.9% (*n* = 443) were discharged as non-recovered, 0.7% (*n* = 104) were transferred to inpatient services, and 0.2% (*n* = 27) were known (died, to be dead or to have passed) during the treatment episode. The program is meeting SPHERE minimum standards with a cure rate of greater than 75%, defaulter rate less than 15%, and mortality rate less than 5% [16]. Figure 5 shows the outcomes (as proportions of all exits) for participants in this program as a standard program monitoring chart.

The multivariate analysis found that those who travel long distances to reach to the treatment centers, or having diarrhea, vomiting, fever, or cough during the treatment episode had increased odds of experiencing a negative treatment outcome (i.e. of not being discharged as cured). Those with higher MUACs at admission, having diarrhea or cough at admission had decreased odds of experiencing a negative treatment outcomes [Table 7].

**Table 7:** Multivariable analysis associated with negative outcomes in the sample of admissions from the Nigeria Outpatient Therapeutic Program (2010 to 2013)

Variable	Strength of association^a^
Time-to-travel^b^	1.08 (1.07;1.10)
MUAC at admission^c^	0.96 (0.96;0.96)
Diarrhea at admission	0.87 (0.83;0.91)
Cough at admission	0.88 (0.83 0.93)
Diarrhea during the treatment episode	1.51 (1.37;1.66)
Vomiting during the treatment episode	1.73 (1.49;2.00)
Fever during the treatment episode	1.28 (1.15;1.42)
Cough during the treatment episode	1.38 (1.21;1.58)

^a^Results presented as adjusted odds ratio (AOR) and 95% confidence interval

^b^AOR is for an increase of one hour in travel time

^c^AOR is for an increase of 1 mm in MUAC

## Discussion

This is a large-scale CMAM program operating in Nigeria. The findings from the most recent data in the better performing state program show that 87.1% of admitted cases were discharged as cured. The program is meeting SPHERE minimum standards with a cure rate above 75%, defaulter rate below 15%, and mortality rate below 5% [16]. The SPHERE minimum standards are usually applied to well-resourced emergency programs operating with considerable United Nation Organization (UNO), Non-Governmental Organization (NGO), and donor support. For a national program to meet these standards is, therefore, something of an achievement.

Previously, CMAM programs used a two-stage approach for screening and admission procedure. This used MUAC (at a high threshold such as 135 mm to ensure adequate case-finding sensitivity) for screening and referral of SAM cases at a community level by community-based volunteers and used weight-for-height at health facilities level to decide admission. This led to large numbers of children referred to the CMAM program being refused treatment because they do not meet the weight-for-height admission criterion [8, 17]. Rejected children often did not attend the program even when their nutritional status declined. Rejection also acted to discourage attendance in new referrals. This had a powerful negative impact upon program coverage [18]. To avoid this “problem of rejected referrals” [8, 17], MUAC is increasingly used to identify and admit children with acute malnutrition for treatment. Studies have shown that MUAC < 115 mm identifies more severely malnourished children with a high risk of mortality if untreated than WHZ [19] and that there is no benefit in using both WHZ less than − 3 and / or MUAC less than 115 mm for admission purpose.

Coverage assessments using the Semi-Quantitative Evaluation of Access and Coverage (SQUEAC)/Simplified Lot Quality Assurance Sampling Evaluation of Access and Coverage (SLEAC) methodologies [20, 21] of the Nigerian CMAM program have yielded coverage estimates of about 38% (2013) and 37% (2014). These results are below SPHERE minimum standards for coverage but are not exceptional. Rogers et al. (2015) examined the coverage of 44 emergency CMAM programs from 21 countries and found an average coverage of 38.3% with 38 of the 44 studied programs failing to meet SPHERE minimum standards [18]. The Nigerian CMAM program is delivering similar, admittedly substandard, coverage to that being achieved in other settings.

The median age at admission in this study was 13 months which is similar to studies carried out in Burkina Faso [22] and Sudan [23]. This is a reflection of the peak age of children at which many suffer from acute respiratory infection (ARI) and diarrheal episodes [24, 25].

The average weight velocity in this study was 3.4 g/kg/day. This is similar to that reported elsewhere [22, 26, 27]. In our study, it was also found that the length of stay for those recovered children was 7 weeks / 49 days which is lower than the study in Gedaref, Northern Sudan which showed 60 days [23], 54 days in Burkina Faso [22] and higher than 42 days reported from Myanmar [27]. These differences might be attributed to the different discharge criteria used in the different settings, which can influence the length of stay. In Northern Sudan, MUAC ˃ 125 mm for 2 consecutive measurements with stable weight or continuing weight gain was used while 15% weight gain and a minimum length of stay of 4 weeks was used for discharge in Burkina Faso.

According to the guidelines for selective feeding programs for the management of malnutrition in emergencies by UN High Commissioner for Refugees (UNHCR) / the World Food Programme (WFP) in collaboration of the United Nations Standing Committee on Nutrition(UNSCN) and the WHO, the standard average length stay for recovered children in therapeutic programme should be below 60 days for children in inpatient and outpatient care combined [28]. The protocols for monitoring weight gain and clinical condition should ensure that the child reaches discharge criteria in approximately 8 weeks [11]. Therefore, our finding support that the evidence that MUAC admission and discharging is possible whilst maintaining reasonable lengths of stay in the program with adequate weight gain.

In this study, factors associated with negative outcomes were distance between home and treatment centers [OR = 1.08; (95% = 1.07; 1.10)], lower MUAC [OR = 0.96; 95% = (0.96;0.96)], diarrhea[OR = 0.87,95% = (0.83;0.91)] and cough[OR = 0.88, 95% = (0.83 0.93)] at admission or having diarrhea[OR = 1.51;95% = (1.37;1.66)], vomiting[OR = 1.73; 95% = (1.49;2.00)], fever [OR = 1.28; 95% = (1.15;1.42)]or cough[OR = 1.38;95% = (1.21;1.58)] during the treatment episode. For these identified problems, we suggest practical activities and interventions that should help address these issues in the Nigerian CMAM program [Table 8].

**Table 8:** Reasons for negative outcomes and proposed possible suggestions

Possible reasons for negative outcome	Practical solution suggested
• Distance between home and treatment centres	• Providing CMAM services directly in a greater number of communities using community based health workers (CHWs) or health extension workers (HEWs) to deliver CMAM services in their own communities.
• Lower MUAC at admission	• Late treatment seeking (i.e. lower MUAC at admission) is usually associated with high opportunity costs [31]. These can be reduced by reducing time-to-travel / distance. • Allowing mothers / caretakers to screen their own children to identify malnutrition early, facilitating referral and admission into malnutrition treatment program is recommended so as to reduce cost and decrease the rate of hospitalization [31, 32].
• Having diarrhoea, vomiting, fever and cough during the treatment episode that reduced response to treatment.	• Counseling of mothers by clinic staff and community-based volunteers regarding the importance of early treatment seeking for conditions such as diarrhoea, vomiting, fever, and cough at any time and especially during the treatment episode. • Counseling mothers on appropriate treatment (i.e. ORS) for conditions such as diarrhoea and vomiting. • Delivery and adherence to the full CMAM protocol to all cases while under treatment in program sites • Enhanced clinical screening of all beneficiaries at each visit to facilitate early detection of comorbidities. A two-stage screen employing a simple formal question-set administered by lower-level clinic staff followed by a clinical screen by a nurse or doctor is likely to prove the most cost-effective approach. • Provision of effective (i.e. second or third line) antimicrobials to all children with infections needing treatment

The study has strength and limitations. The strength of the study is having large sample size nature of the data that captured in the study and as MUAC is particularly suitable for large scale studies and surveys, as it can be measured with limited resources for human population surveys, especially among rural populations of developing countries [29]. However, some incomplete data has been reported due to secondary nature of the data taken from routine program in health facilities.

## Conclusions

This study confirms that MUAC can be used for both admission and discharge criteria in CMAM programs. Long distance between home and treatment centers, lower MUAC, diarrhea and cough at admission, or having diarrhea, vomiting, fever or cough during the treatment episode were associated with negative outcomes. Providing CMAM services closer to the community using either mobile clinics or satellite clinics or increasing the number of health facilities delivering CMAM services should minimize distance to travel. Conducting community sensitization and mobilization activities, counseling of mothers by health workers about early treatment seeking behavior and screening of cases for early detection of comorbidities is recommended. Identifying and addressing reasons for negative outcome is essential to achieving the goals of CMAM at the primary health care facilities.

## References

[1] Caulfield L, MdO M, Blössner M, Black R (2004). Undernutrition as an underlying cause of child deaths associated with diarrhea, pneumonia, malaria, and measles. Am J Clin Nutr.

[2] Bhutta Z, Ahmed T, Black R, Cousens S, Dewey K, Giugliani E, Haider B, Kirkwood B, Morris S, Sachdev H (2008). What works? Interventions for maternal and child undernutrition and survival. Lancet.

[3] Lapidus N, Minetti A, Djibo A, Guerin P, Hustache S, Gaboulaud V, Grais R (2009). Mortality risk among children admitted in a large-scale nutritional program in Niger, 2006. PLoS One.

[4] Black R, Allen L, Bhutta Z, Caulfield L, Onis Md, Ezzati M, Mathers C, Rivera J: Maternal and child undernutrition: global and regional exposures and health consequences. Lancet 2008, 371(9608):243–260. 10.1016/S0140-6736(07)61690-0 18207566

[5] Collins S (2001). Changing the way we address severe malnutrition during famine. Lancet.

[6] WHO UNICEF (2009). WHO child growth standards and the identification of severe acute malnutrition in infants and children.

[7] UNICEF/NBS: Summary findings of National Nutrition and health survey using SMART methods. United Nations children funds (UNICEF)/Nigeria National Bureau of statistics (NBS) 9th Feb to 5th may, Nigeria. 2014.

[8] Myatt M, Khara T, Collins S (2006). A review of methods to detect cases of severely malnourished children in the community for their admission into community-based therapeutic care programs. Food Nutr Bull.

[9] Hamer C, Kvatum K, Jeffries D, Allen S (2004). Detection of severe protein-energy malnutrition by nurses in the Gambia. Arch Dis Child.

[10] Velzeboer M, Selwyn B, Sargent F, Pollitt E, Delgado H (1983). The use of arm circumference in simplified screening for acute malnutrition by minimally trained health workers. J Trop Pediatr.

[11] FMOH: National operational guidelines for community management of acute malnutrition(CMAM). Federal Ministry of Health. August 2010.

[12] FMOH: Operational guidelines for community management acute malnutrition (CMAM) in Nigeria. Federal Ministry of Health. August 2010.

[13] Lauritsen J: EpiData Data Entry, Data Management and basic Statistical Analysis System. Odense Denmark, EpiData Association, Available at www.epidata.dk 2000–2008. Accessed 24 July 2014.

[14] R: Core Team R: A language and environment for statistical computing. R Foundation for Statistical Computing. Vienna, Austria 2014.

[15] Ellis P (2010). The essential guide to effect sizes: statistical power and the interpretation of research results.

[16] The Sphere Project. Humanitarian Charter and Minimum Standards in Humanitarian. 2011. 10.1111/j.0361-3666.2004.00245.x 20958782

[17] Guerrero S, Myatt M, Collins S (2010). Determinants of coverage in community-based therapeutic care programmes: towards a joint quantitative and qualitative analysis. Disasters.

[18] Rogers E, Myatt M, Woodhead S, Guerrero S, Alvarez J (2015). Coverage of community-based management of severe acute malnutrition programmes in twenty-one countries, 2012-2013. PLoS One.

[19] Grellety E, Krause L, Eldin MS, Porten K, Isanaka S. Comparison of weight-for-height and mid-upper arm circumference (MUAC) in a therapeutic feeding programme in South Sudan: is MUAC alone a sufficient criterion for admission of children at high risk of mortality? Public Health Nutr. 2015;18(14):2575–81. 10.1017/S1368980015000737 PMC10271431 25805273

[20] Chrissy Banda BS, Safari Balegamire, Mous Bauchi, Ernest Guevarra, Lio Fieschi. Simplified lot quality assurance sampling evaluation of access and coverage (SLEAC) survey of Community Management of Acute Malnutrition program in northern states of Nigeria. Valid international and coverage monitoring Network 2014.

[21] Njau J, Maduanusi I, Bimba E: Semi-quantitative evaluation of access and coverage in Jigawa state, Nigeria. Coverage monitoring Network 2013.

[22] Goossens S, Bekele Y, Yun O, Harczi G, Ouannes M, Shepherd S (2012). Mid-upper arm circumference based nutrition programming: evidence for a new approach in regions with high burden of acute malnutrition. PLoS One.

[23] Dale N, Myatt M, Prudhon C, AB A (2013). Using mid-upper arm circumference to end treatment of severe acute malnutrition leads to higher weight gains in the most malnourished children. PLoS One.

[24] Lima A, Moore S, Barboza M, Soares MJ, Schleupner M, Newman R, Sears C, Nataro J, Fedorko D, Wuhib T (2000). Persistent diarrhea signals a critical period of increased diarrhea burdens and nutritional shortfalls: a prospective cohort study among children in northeastern Brazil. J Infect Dis.

[25] Martorell R, Young M (2012). Patterns of stunting and wasting: potential explanatory factors. Adv Nutr.

[26] Manary M, Ndkeha M, Ashorn P, Maleta K, Briend A (2004). Home based therapy for severe malnutrition with ready-to-use food. Arch Dis Child.

[27] James P, NVd B, Rozet A, Israël A, Fenn B, Navarro-Colorado C (2015). Low-dose RUTF protocol and improved service delivery lead to good programme outcomes in the treatment of uncomplicated SAM: a programme report from Myanmar. Matern Child Nutr.

[28] UNHCR/WFP/WHO: Guidelines for selective feeding: the management of malnutrition in emergencies. Geneva: UNHCR; 2011. Available: http://www.unhcr.org/publications/operations/4b7421fd20/guidelines-selective-feeding-management-malnutrition-emergencies.html. Accessed 20 Jan 2015.

[29] Sultana T, Karim M, Ahmed T, Hossain M (2015). Assessment of under nutrition of Bangladesh adults using anthropometry: can body mass index be replaced by mid-upper-arm-circumference?. PLoS One.

[30] Cohen J (1988). Statistical power analysis for the behavioral sciences.

[31] Blackwell N, Myatt M, Allafort-Duverger T, Balogoun A, Ibrahim A, Briend A (2015). Mothers understand and can do it (MUAC): a comparison of mothers and community health workers determining mid-upper arm circumference in 103 children aged from 6 months to 5 years. Arch Public Health.

[32] Alé FGPK, Issa H, Defourny I, Le Duc G, Harczi G, Issaley K, Sayadi S, Ousmane N, Yahaya I, Myatt M, Briend A, Allafort-Duverger T, Shepherd S, Blackwell N (2016). Mothers screening for malnutrition by mid-upper arm circumference is non-inferior to community health workers: results from a large-scale pragmatic trial in rural Niger. Arch Public Health.

