# Correction to: Mid-upper-arm circumference based case-detection, admission, and discharging of under five children in a large-scale community-based management of acute malnutrition program in Nigeria

**DOI:** 10.1186/s13690-018-0271-7

**Published:** 2018-06-28

**Authors:** Stanley Chitekwe, Sibhatu Biadgilign, Assaye Tolla, Mark Myatt

**Affiliations:** 1 United Nations Children’s Fund (UNICEF), Nigeria country office, UN House, Plot 617/618 Central Area District Diplomatic Zone, Garki, Abuja B 2851 Nigeria; 2 Consultant Epidemiologist, Brixton Health, Llawryglyn, Wales, UK

## Correction

The author requests to correct errors in the original article [1]. Area where it needs amendment are as follows.

1. In conclusion section of Abstract:

“This study confirms that MUAC can be used for both admitting and discharging criteria in CMAM programs with MUAC < 115 mm for admission and MUAC > 125 mm or at discharge (a higher discharge threshold could be used).” 

Replaces

“This study confirms that MUAC can be used for both admitting and discharging criteria in CMAM programs with MUAC < 115 mm for admission and MUAC > = 115 mm or at discharge (a higher discharge threshold could be used).”

2. In Table 1:

1. All “MUAC > 115 mm” should be changed into “MUAC > 125 mm” under each of the discharge criteria column.
2. Superscript “a” above the “well” should be removed.
3. The legend under the table should be removed.
